# Effect of *Pholiota nameko* Polysaccharides Inhibiting Methylglyoxal-Induced Glycation Damage In Vitro

**DOI:** 10.3390/antiox10101589

**Published:** 2021-10-10

**Authors:** His Lin, Ting-Yun Lin, Jer-An Lin, Kuan-Chen Cheng, Shella Permatasari Santoso, Chun-Hsu Chou, Chang-Wei Hsieh

**Affiliations:** 1 Department of Food Science and Biotechnology, National Chung Hsing University, 145 Xingda Rd., South Dist., Taichung City 40227, Taiwan; xixi104008703@gmail.com (H.L.); t6i3n9a0@gmail.com (T.-Y.L.); 2 Graduate Institute of Food Safety, National Chung Hsing University, 145 Xingda Rd., South Dist., Taichung City 40227, Taiwan; lja@nchu.edu.tw; 3 Institute of Biotechnology, National Taiwan University, Taipei 10617, Taiwan; kccheng@ntu.edu.tw; 4 Graduate Institute of Food Science Technology, National Taiwan University, Taipei 10617, Taiwan; 5 Department of Optometry, Asia University, 500, Lioufeng Rd., Wufeng, Taichung City 41354, Taiwan; 6 Department of Medical Research, China Medical University Hospital, Taichung City 406040, Taiwan; 7 Department of Chemical Engineering, Widya Mandala Surabaya Catholic University, Kalijudan 37, Surabaya 60114, Indonesia; shella@ukwms.ac.id; 8 Department of Chemical Engineering, National Taiwan University of Science and Technology, Keelung Rd. 43, Da’an Dist., Taipei 10607, Taiwan; 9 Dr Jou Biotech Co., Ltd., No. 21, Lugong S. 2nd Rd., Lukang Township, Changhua 505, Taiwan; dr.jason.jou@gmail.com

**Keywords:** antiglycation, advanced glycation end products, glycation stress, *Pholiota nameko*, polysaccharide, human dermal fibroblasts, cell aging

## Abstract

Advanced glycation end products (AGEs) can induce oxidative stress and inflammation. AGEs are major risk factors for the development of many aging-related diseases, such as cancer and diabetes. In this study, *Pholiota nameko* polysaccharides (PNPs) were prepared from water extract of *P. nameko* via graded alcohol precipitation (40%, 60%, and 80% *v*/*v*). We explored the in vitro antiglycation ability of the PNPs and inhibition of methylglyoxal (MG)-induced Hs68 cell damage. In a bovine serum albumin (BSA) glycation system, PNPs significantly inhibited the formation of Amadori products. Fluorescence spectrophotometry revealed that the PNPs trapped MG and reduced MG-induced changes in functional groups (carbonyl and ε-NH_2_) in the BSA. Pretreating Hs68 cells with PNPs enhanced the cell survival rate and protected against MG-induced cell damage. This was due to decreased intracellular ROS content. PNPs thus mitigate skin cell damage and oxidative stress resulting from glycation stress, making them a potential raw material for antiaging-related skincare products.

## 1. Introduction

Glycation is a nonenzymatic reaction that occurs between sugars and proteins [1], such as glucose, and other macromolecules including proteins, nucleic acids, and lipids. Through condensation and rearrangement, a series of complex chemical reactions, such as cleavage and oxidative modification, eventually produces a group of structurally stable compounds called advanced glycation end products (AGEs). High sugar concentration or impaired carbohydrate metabolism can cause overproduction of AGEs and damage to physiological functions. This effect is called glycation stress. Recent studies have confirmed that glycation stress is related to the development and complications of various diseases, such as diabetes, cardiovascular disease, obesity, kidney disease, and neurological and cognitive-related diseases [2,3,4,5,6,7,8]. Glycation stress also plays a critical role in the aging process because extreme accumulation of AGEs in the body can exacerbate the degradation of physiological functions during aging by increasing oxidative stress and inflammation [9].

The skin, as the organ with the largest surface area, protects the human body from the environment in addition to serving other important physiological functions [10]. Fibroblasts are the main cells in the dermis and have significant importance in the process of skin aging [11]. Human skin changes in appearance not only due to the aging process but also from the development of disease. Researchers have recently been focusing on the relationship between glycation stress and skin aging. Due to the long half-life of elastin and collagen in the skin, it is prone to glycation reactions with AGEs, resulting in crosslinking. The protein deforms, stiffens, and eventually loses its function, which exacerbates the decline in elasticity and appearance of wrinkles during the aging process. Additionally, the combination of AGEs in the skin and the specific receptor RAGE promotes the secretion of inflammatory factors, and intracellular signal conduction may cause skin cell apoptosis and lower the metabolism rate during the aging process [12]. Hence, many studies have investigated naturally derived products that can slow skin aging by inhibiting glycation stress.

*Pholiota nameko* is a species of nutritious mushroom originating in Japan [13]. It is rich in protein, carbohydrates, fiber, vitamins, and unsaturated fatty acids [14]. Polysaccharides are the main active constituents in the fruiting bodies of *P. nameko*. Many studies have confirmed that the polysaccharides of *P. nameko* have antioxidative, anti-inflammatory, hypolipidemic, and antiaging effects [15,16,17]. In in vitro experiments, a previous study showed that graded alcohol precipitation of *P. nameko* polysaccharides (40% *v*/*v*, PNP-40; 60% *v*/*v*, PNP-60; 80% *v*/*v*, PNP-80) revealed antioxidant abilities, such as scavenging ABTS and DPPH free radicals, chelating metal ferrous ions, and protecting L929 cells against oxidative damage induced by H_2_O_2_, as well as promoting cell migration and proliferation [18].

Many recent studies have focused on developing naturally derived substances with antioxidant potential as therapeutic agents [19,20,21,22,23]. The antiglycation ability of polysaccharides has received particular attention. Polysaccharides isolated and purified from *Boletus snicus*, *Ribes nigrum* L., and *Actinidia arguta* have demonstrated favorable in vitro antioxidant and antiglycation effects. Studies have shown that the antiglycation mechanism in naturally sourced polysaccharides is mostly related to their antioxidant capacity and composition [24,25]. Therefore, the present study evaluated the in vitro antiglycation efficacy of PNPs using the MG–BSA model and their ability to protect human fibroblast cells from damage under glycation stress. These PNPs could be developed as a potential raw material in cosmetics or medicines industries.

## 2. Materials and Methods

### 2.1. Chemicals

Methylglyoxal (MG); bovine serum albumin (BSA); aminoguanidine (AG) hemisulfate salt; o-phenylenediamine; nitroblue tetrazolium (NBT); 2,4,6-trinitrobenzenesulphonic acid (TNBS); 2,4-dinitrophenylhydrazine (DNPH); 5,5′-dithiobis-(2-nitrobenzoic acid) (DTNB); sodium dodecyl sulfate; *N*,*N*,*N*′,*N*′,-tetramethylethylenediamine; and Coomassie brilliant blue G-250 were purchased from Sigma-Aldrich (Sigma-Aldrich, St. Louis, MO, USA). HPLC methyl alcohol and HPLC acetic acid were purchased from Daejung (Daejung, Gyeonggi-do, Korea).

### 2.2. Sample Preparation

*P. nameko* was purchased from the Rich Year Farm (Puli Township, Nantou County, Taiwan). PNPs were prepared as described in previous research [13]. Briefly, the PNPs were extracted using hot water and precipitated via graded ethanol precipitation. Ethanol was added at final concentrations of 40% *v/v* (PNP-40), 60% *v/v* (PNP-60), and 80% *v/v* (PNP-80). All samples were collected, lyophilized, and then refrigerated at 4 °C.

### 2.3. MG–BSA Glycation Model

Our MG–BSA glycation system was adapted from previous research [26]. A 0.1 M phosphate buffer solution (pH 8.0) was used to configure the MG (system concentration: 25 mM) with BSA (5 mg/mL). BSA solution without MG was used as a control. The reaction was maintained at 37 °C for 24 h.

#### 2.3.1. Inhibition Effects on the Formation of Amadori Products

Amadori product content was determined in order to evaluate the ability of PNPs to inhibit Amadori product production in the early stage of saccharification. The MG–BSA group and the group with PNPs (final concentrations of 0.5, 1.0, and 1.5 mg/mL) were measured.

Test samples (20 µL) were mixed with 0.25 mM NBT in a 100 mM sodium carbonate buffer (pH 10.35) and reacted at 37 °C for 2 h. After the reaction, absorbance was measured at 525 nm with an enzyme immunoassay reader (Thermo Scientific™ 51119200 microplate spectrophotometer, Waltham, MA, USA) [27].

(1) $$\mathrm{Amadori}\;\mathrm{product}\;\mathrm{content}\;(\mathrm{fold}\;\mathrm{of}\;\mathrm{control})=\frac{OD_{570\left(MG+BSA\right)}-OD_{570\left(control\right)}}{OD_{570\left(control\right)}}$$

#### 2.3.2. MG-Trapping

The experimental design was adapted from a previous study [28]. A high-performance liquid chromatograph (HPLC; Hitachi, Tokyo, Japan) was employed to analyze the ability of lutein to capture the metaphase dicarbonyl compound MG. For each grade, 0.5 mL of different concentrations of PNPs (0.5, 1, and 1.5 mg/mL) was dissolved in water and mixed with 0.5 mL of 2 mM MG and 0.5 mL of 12 mM o-phenylenediamine. Samples were then reacted at 37 °C for 30 min for derivatization. Analysis was performed after filtering with a 0.22 μm syringe filter. The HPLC system (Hitachi 5110 pump, Hitachi 5260 Auto Sampler, Hitachi 5260 auto-degasser, and Hitachi 5420 UV-VIS detector, Tokyo, Japan) was equipped with a C18 column (250 nm × 4.6 mm, ID: 5 µm, Code No.: 38145-21, Nacali Tesque). The column was flushed with a mixture of 4:60.15% acetic acid/water–methanol at a flow rate of 0.8 mL/min. The injection volume was 20 μL, and the wavelength used for detection was 315 nm. AG was employed as a positive control to compare the residence time of the analysis peak of the standard and the sample and to calculate the ratio of the peak area of the sample relative to the peak area of the standard. The MG-trapping percentage was calculated using the following formula:

(2) $$\mathrm{MG-trapping}\;(\%)=[100-\frac{Amount\;of\;MG_{\left(sample\right)}-\;Amount\;of\;MG_{\left(control\right)}}{\;Amount\;of\;MG_{\left(control\right)}}]\times 100\%$$

#### 2.3.3. Inhibition Effects on the Formation of Carbonyl Groups

Following previous research but with some modification [29], the MG–BSA system was incubated at 37 °C for 3 days. Then, 100 µL of the test solution was added to 400 µL of 2 mM DNPH (prepared with a 2N HCl solution) and reacted for 1 h at room temperature with vortexing every 15 min during the reaction. After the reaction was completed, 0.5 mL of 20% TCA was added and centrifuged at 13,000× *g* at 4 °C. After removal of the supernatant, the protein precipitate was collected. The precipitate was washed three times using EA/EtOH (1:1), after which 0.3 mL of 6 M guanidine solution was added to it. Finally, the precipitate was incubated in a water bath at 37 °C for 15 min. After the reaction was completed, the sample was measured using an enzyme immunoassay reader (Thermo Scientific^TM^ 51119200 microplate spectrophotometer, Waltham, MA, USA), with the absorbance measured at 360 nm.

(3) $$\mathrm{Carbonyl}\;\mathrm{product}\;\mathrm{content}\;(\mathrm{fold}\;\mathrm{of}\;\mathrm{control})=\frac{OD_{360\left(MG+BSA\right)}-OD_{360\left(control\right)}}{OD_{360\left(control\right)}}$$

#### 2.3.4. Inhibition Effects on the Decrease in ε-NH_2_ Group

Based on previous research [30], the MG–BSA system was incubated at 37 °C for 3 days. Next, 500 µL of the test solution was added to 100 µL of 0.5% TNBS solution and incubated in a water bath at 37 °C for 1 h. After the reaction was completed, 0.25 mL of SDS and 0.1 mL of HCL were added. Samples were then measured using the enzyme immunoassay reader, and the absorbance was measured at 420 nm.

(4) $$\varepsilon -NH_{2}\;\mathrm{product}\;\mathrm{content}\;(\mathrm{fold}\;\mathrm{of}\;\mathrm{control})=\frac{OD_{420\left(MG+BSA\right)}-OD_{420\left(control\right)}}{OD_{420\left(control\right)}}$$

#### 2.3.5. Sodium Dodecyl Sulfate Polyacrylamide Gel Electrophoresis

Follow previous research [31], the MG–BSA system was incubated at 37 °C for 5 days. The test solution was then mixed and diluted with protein staining dye, after which it was heated at 95 °C on a hot plate for 5 min. Protein samples were separated by 10% SDS–PAGE (pressing the gel at 70 V for 15 min and then running the gel at 140 V for 1 h). The film was washed three times with a TBST buffer and then dyed using 0.125% Coomassie blue. After 3 h, a destaining agent (65% dd H_2_O + 25% MetOH + 10% acetic acid (*v*/*v*)) was applied for 3 h, and images were captured using a camera system (Biospectrum 810 Imaging system, Fisher Scientific, Upland, CA, USA).

#### 2.3.6. Fluorescent AGE Analysis

The MG–BSA system was incubated at 37 °C for 5 days. Test solutions (1 mL) were then diluted with dd H_2_O five times and scanned via fluorescence spectrophotometry (start WL: 200 nm, end WL:550 nm, scan speed: 12,000 nm/min) [32].

### 2.4. Cell Culture

Human dermal fibroblast cell line Hs68 (Obtained from ATCC CRL-1635, Manassas, VA, USA) was cultured in Dulbecco’s modified Eagle’s medium (DMEM, Gibco^®^, Grand Island, NY, USA) containing 10% fetal bovine serum (FBS, Gibco^®^, Grand Island, NY, USA) and antibiotics (100 U penicillin and 100 U/mL streptomycin, Gibco^®^, Grand Island, NY, USA) under 5% CO_2_ at 37 °C. Cells were harvested after reaching confluence by using 0.05% trypsin–EDTA (Gibco^®^, Grand Island, NY, USA). Fresh culture medium was added to produce single-cell suspensions for further incubation.

### 2.5. Cell Viability

Cell viability was determined using an MTT assay, following a previously described procedure with slight modification [13,18]. Hs68 cells were seeded in 96-well plates (5 × 10^3^ cells/well) and allowed to adhere for 24 h. The cells were incubated with 200 µL of DEME containing PNPs at concentrations of 0.5, 1.0, and 1.5 mg/mL. Cells without PNPs were used as controls. After incubating for 24 h, 3-[4,5-dimethyl thiazol-2-yl]-2,5-diphenyltetrazolium bromide was dissolved in 1 × PBS to prepare the MTT stock solution (5 mg/mL), which was then diluted with DMEM. Next, 100 µg/mL MTT solution was added to the 96-well plates and incubated under 5% CO_2_ at 37 °C for 2 h, after which 200 μg/mL dimethyl sulfoxide (DMSO) was added to each well. Absorbance was measured at 570 nm using an ELISA reader. Cell viability was calculated using the following equation:

Cell viability (%) = [(*OD*_570_(*sample*)/*OD*_570_(*control*)] × 100% (5)

where *OD*_570_ (*sample*) is the absorbance of the sample, and *OD*_570_ (*control*) is that of the control.

### 2.6. MG-Induced Cell Oxidative Damage

#### 2.6.1. Determination of Protective Ability of PNPs against MG-Induced Cell Damage

The MG-induced cell damage model was constructed similarly to previous research but with minor modifications [33]. First, 5 × 10^3^ cells/well of Hs68 cells were seeded in 96-well plates for 24 h. After the cells had adhered to the plates, they were treated with PNPs (0.5, 1.0, and 1.5 mg/mL) for 12 h. Residual medium was removed by washing the cells twice with 1 × PBS, after which the cells were treated with MG (400 µM) for 24 h. The cells were then washed twice again with 1 × PBS. Then, 100 µL of MTT solution (500 µg/mL each well) was added, and the cells were left to incubate for 2 h. To evaluate the cell viability, 200 µL of DMSO was added to the samples. Absorbance was measured at 570 nm.

#### 2.6.2. MG-Induced Cell Growth Rate Assay

This experiment was designed based on previous studies but with minor modifications [34]. First, 5 × 10^4^ cells/well of Hs68 cells were seeded in 12-well plates for 12 h. After the cells had adhered to the plates, they were treated with PNPs (0.5, 1.0, and 1.5 mg/mL) for 12 h. The cells were then washed with 1 × PBS twice and treated with MG (400 μM) for 24 h. Next, 200 µL of 1 × trypsin was added to each well for cell subculturing. Cells were counted using an automated cell counter (LUNA-II^TM^, Anyang, Korea) and prediluted to 5 × 10^4^ cells/well for seeding in 12-well plates. A total of 10 generations of cell subcultures were prepared for the experiment.

#### 2.6.3. ROS Generation

The ROS generation assay was performed using a previously described method, with modification [18]. Specifically, the concentration was evaluated using a DCF-DA probe (Sigma-Aldrich, St. Louis, MO, USA). Hs68 cells were seeded in 6-well plates at a concentration of 8 × 10^5^ cells/well and allowed to adhere for 24 h. The cells were incubated with PNPs (0.5–1.5 mg/mL) for 12 h and then treated with MG (400 μM) for 24 h. After treatment, the Hs68 cells were incubated in DMEM (without FBS) containing DCF-DA (10 μM) at 37 °C in the dark for 30 min. The probe was then removed and washed twice in PBS. The final results were evaluated using a fluorescence microscope (Olympus IX51, Tokyo, Japan). The mean density values of the captured images were analyzed using Image J software (ImageJ, Bethesda, MD, USA).

### 2.7. Statistical Analysis

All data are expressed as the mean ± standard deviation (SD). Statistical data processing was implemented through dispersion analysis with SPSS 20 software (IBM Analytics, Chicago, IL, USA). One-way ANOVA and Duncan’s multiple range tests were conducted with *p* < 0.05 considered the level of statistical significance [18].

## 3. Results

### 3.1. Inhibition Effects of PNPs on the Formation of Amadori Products

**Figure 1:**
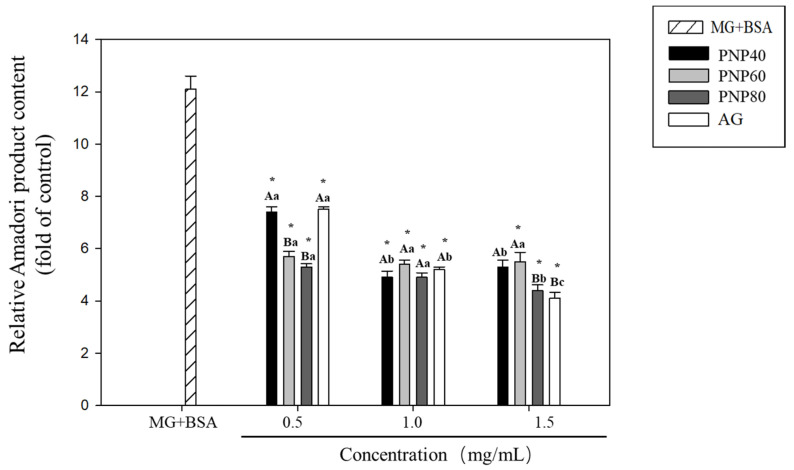
Effect of PNPs on the formation of Amadori products (0.5, 1.0, and 1.5 mg/mL). * Significant difference with the controls; A and B: significant difference between different samples with the same concentration (*p* < 0.05); a–c: significant difference with the same sample at different concentrations (*p* < 0.05).

Figure 1 shows the effect of PNPs on the formation of Amadori products. MG treatment significantly increased the Amadori product content of the BSA (12.1-fold) relative to the controls (*p* < 0.05). However, treatment with all three concentrations of PNP-40, PNP-60, and PNP-80 (i.e., 0.5, 1.0, and 1.5 mg/mL) significantly reduced the MG-induced Amadori products (*p* < 0.05). The fold increases observed in PNP-40 were 7.4, 4.9, and 5.3 for 0.5, 1.0, and 1.5 mg/mL, respectively, with corresponding inhibition rates of 39%, 59.9%, and 56.3%. For PNP-60, the fold increases were reduced to 5.7, 5.4, and 5.5, respectively, with inhibition rates of 53.3%, 55.5%, and 54.2%. Finally, for PNP-80, the fold increases were reduced to 5.3, 4.9, and 4.4, respectively. The corresponding inhibition rates were 61.6%, 55.1%, and 63.7%, respectively. While all PNPs inhibited the formation of Amadori products, no significant difference was observed between the different concentrations. Thus, the effect was not considered dose-dependent. Moreover, the inhibitory effect of PNPs was better than the AG inhibitory rate of 37.8% at the lowest concentration (0.5 mg/mL).

### 3.2. MG-Trapping Capacity of PNPs

**Figure 2:**
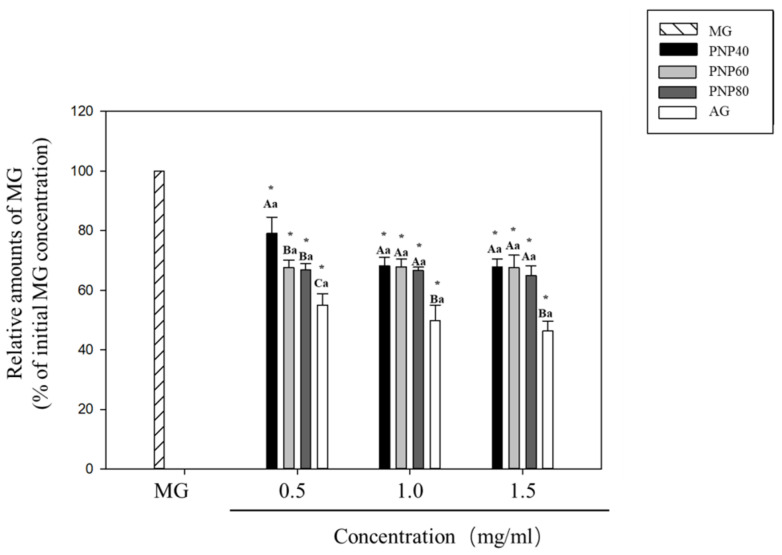
Ability of PNPs to capture MG. MG-relative amounts of MG of different concentrations of PNP-40, PNP-60, PNP-80, and AG (0.5, 1.0, and 1.5 mg/mL). * Significant different with control group; A–C: significant difference between different samples with the same concentration (*p* < 0.05); a: significant difference with the same sample at different concentrations (*p* < 0.05).

Figure 2 shows the MG-trapping capacity of the PNPs. The direct MG-trapping ability was evaluated to investigate whether the PNPs could directly scavenge MG. At concentrations of 0.5–1.5 mg/mL, the MG-trapping ability at 0.5, 1.0, and 1.5mg/mL was 20.9%, 31.9%, and 32.2% for PNP-40; 32%, 32.1%, and 32.4% for PNP-60; and 33.1%, 33.3%, and 34.9% for PNP-80, respectively. For AG, the corresponding rates were 45%, 50%, and 53.6%, respectively.

### 3.3. Inhibition Effects of PNPs on the Formation of the AGEs

**Figure 3:**
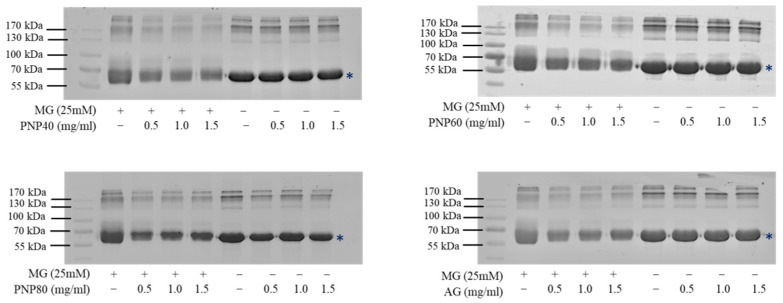
Effects of PNPs and AG (0.5, 1.0, and 1.5 mg/mL) on glycated protein, obtained using SDS–PAGE. The SDS–PAGE profile showed modulation of high molecular weight adduct generation. * BSA molecular weight = 66.5 kDa.

Figure 3 shows that the BSA produced protein bands at 66.2 kDa, indicating that the molecular weight of BSA was unaffected by the PNPs. However, when the BSA (5 mg/mL) and MG (25 mM) were reacted together, the glycated BSA molecular bands on the protein electrophoresis pattern showed obvious upward diffusion (MG + groups). In the PNPs group and positive control group AG (0.5, 1.0, and 1.5 mg/mL), the cross-linking of glycated BSA can be observed as a decrease in aggregation in the protein band.

**Figure 4:**
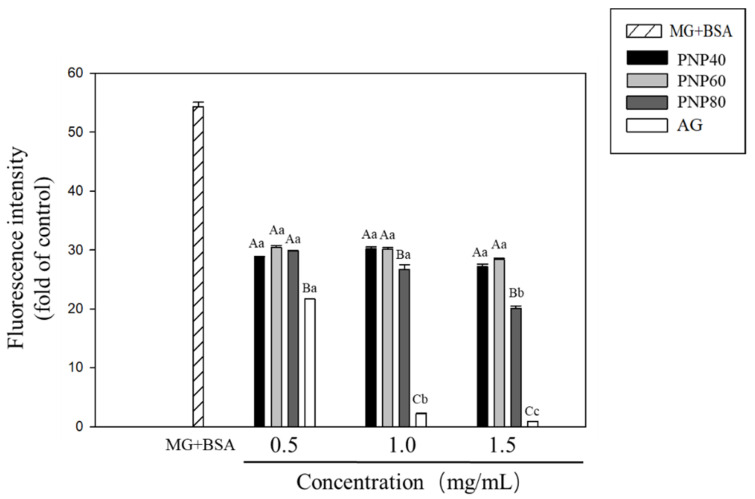
Effects of glycation on the formation of fluorescent AGEs (fluorescence measured at 370 and 440 nm). MG-relative amounts of MG for different concentrations of PNP-40, PNP-60, PNP-80, and AG (0.5, 1.0, and 1.5 mg/mL). A–C: significant difference with the same concentration in different samples (*p* < 0.05); a–c: significant difference with the same sample at different concentrations (*p* < 0.05).

In this experiment, fluorescence analysis further clarified the inhibitory effects of PNPs on protein glycation. Figure 4 shows that the fluorescence signal intensity of the glycation (MG + BSA) group was significantly increased 54.3-fold compared to the controls, with PNPs significantly inhibiting the fluorescence signal induced by MG. At concentrations of 0.5, 1.0, and 1.5 mg/mL, the respective inhibition rates of the glycated fluorescence signals were 47.2%, 44.4%, and 50% for PNP-40; 44.1%, 44.6%, and 47.6% for PNP-60; and 45.1%, 50.9%, and 63.0% for PNP-80. The corresponding rates for AG were 60.1%, 96.0%, and 98.4%, respectively.

### 3.4. Inhibition Effects on the Formation of the Carbonyl Group and Decrease in the ε-NH_2_ Group

**Figure 5:**
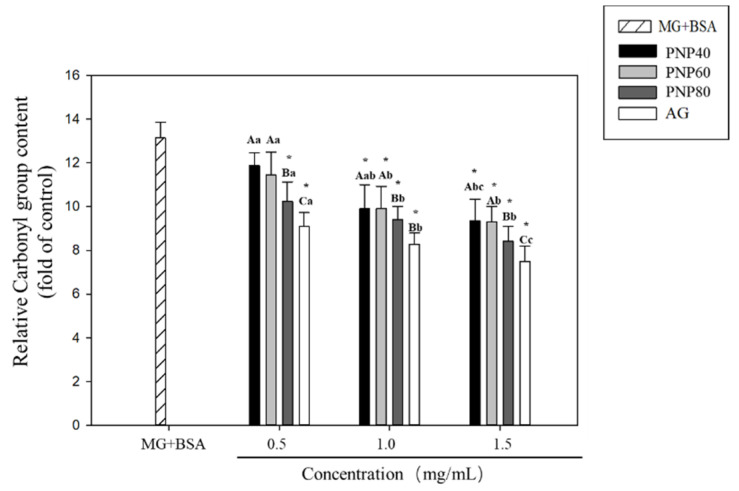
Effect of PNPs on the formation of carbonyl groups (0.5, 1.0, and 1.5 mg/mL). * Significant difference with the controls; A–C: significant difference with the same concentration in different samples (*p* < 0.05); a–c: significant difference with the same sample at different concentrations (*p* < 0.05).

The experimental results revealed that PNPs significantly affected glycation. To clarify the reaction mechanism, this experiment further explored changes of specific functional groups on the glycated protein in the PNP-treated group. Figure 5 shows the results. The carbonyl functional group content of the glycation group was 13.13-fold higher than that of the controls. At concentrations of 0.5, 1.0, and 1.5 mg/mL, the respective inhibition rates were 10%, 24.6%, and 28.8% for PNP-40; 12.8%, 24.6%, and 29.1% for PNP-60; and 22.0%, 28.3%, and 35.9% for PNP-80. For AG, the corresponding rates were 30.6%, 37%, and 42.9%, respectively.

**Figure 6:**
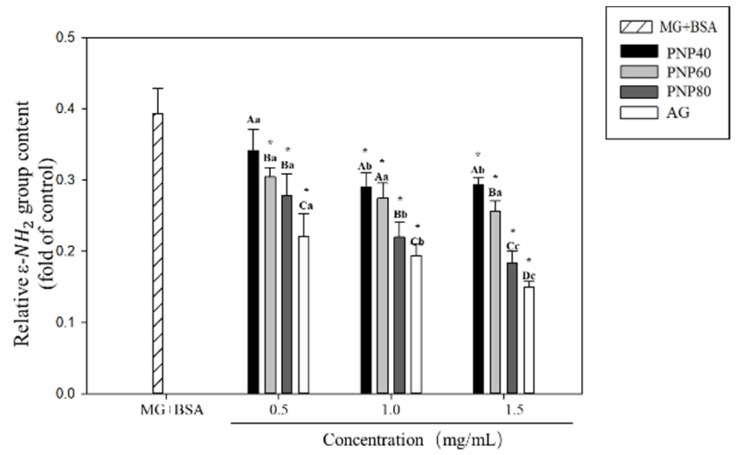
Effect of PNPs on the formation of ε-NH_2_ groups (0.5, 1.0, and 1.5 mg/mL). * Significant difference with the controls; A–C: significant difference with the same concentration in different samples (*p* < 0.05); a–c: significant difference with the same sample at different concentrations (*p* < 0.05).

Figure 6 shows the results that, after glycation, the ε-amine group content in the BSA was significantly reduced by 39.3% relative to the controls, and the PNPs reduced the decrease in the ε-amine group of glycated BSA (*p* < 0.05). At concentrations of 0.5, 1.0, and 1.5 mg/mL, the respective inhibition rates were 12.9%, 23.9%, and 29.3% for PNP-40; 22.4%, 29.9%, and 34.8% for PNP-60; and 28.9%, 43.5%, and 53.1% for PNP-80. For AG, the corresponding rates were 43.9%, 50.7%, and 61.8%. These observations showed that the mechanism of PNPs reducing the AGEs is through significantly reducing the increase in the carbonyl group and decrease in the ε-amine group of the glycated BSA.

### 3.5. Effect of PNPs on Hs68 Cell Viability

**Figure 7:**
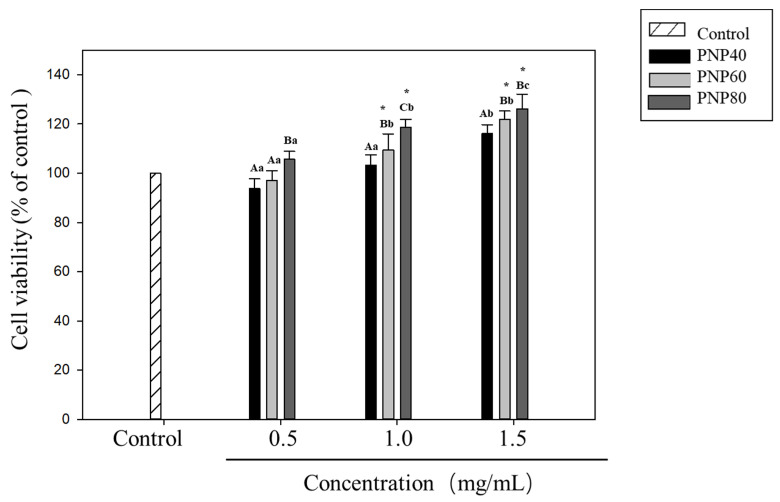
Effect of PNPs on cell viability. Cells were pretreated with PNPs (0.5, 1.0, and 1.5 mg/mL) for 24 h. Viability was determined using an MTT assay. * Significant difference with the control group; A–C: significant difference with the same concentration in different samples (*p* < 0.05); a–c: significant difference with the same sample at different concentrations (*p* < 0.05).

To investigate whether PNPs are toxic to Hs68 cells, cell viability was measured using an MTT assay. Results in Figure 7 are for cells incubated with PNPs for 24 h at concentrations of 0.5, 1.0, and 1.5 mg/mL. At all concentrations, PNP-40, PNP-60, and PNP-80 showed no negative effects on Hs68 cells, while PNP-60 and PNP-80 had a proliferative effect at 1.0 and 1.5 mg/mL.

### 3.6. Effects of PNPs on the Viability of MG-Induced Hs68 Cells

**Figure 8:**
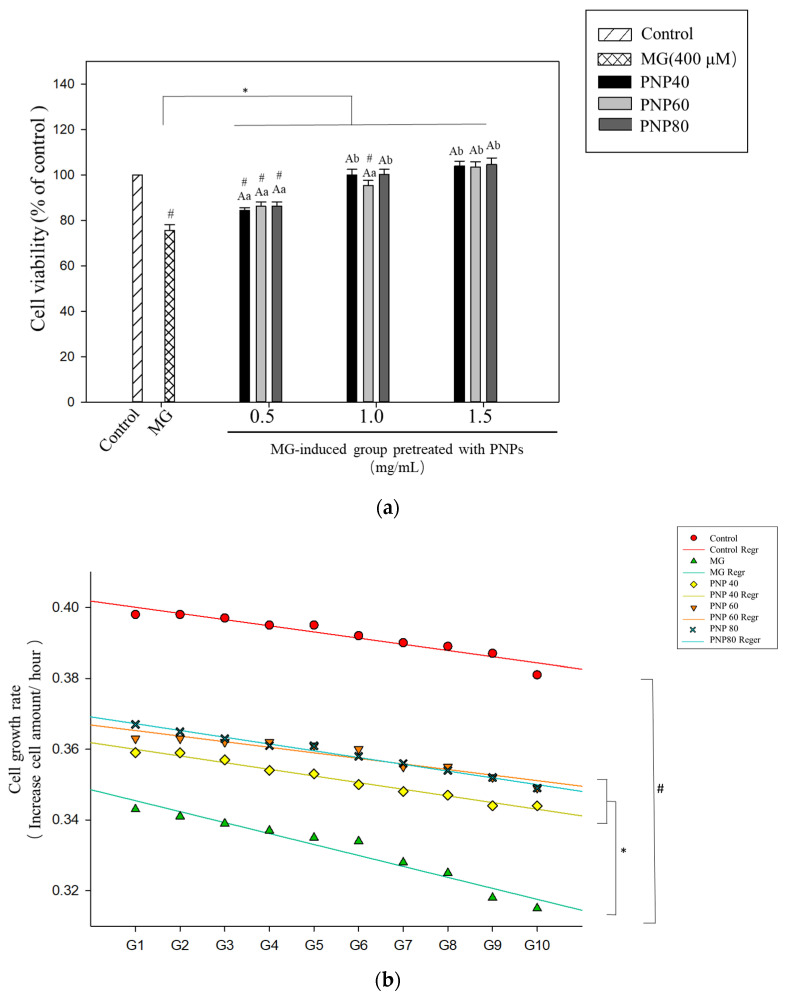
(**a**) PNPs protect Hs68 cells against MG treatment. # Followed by a significant difference with the controls (*p* < 0.05); A: significant difference with the same concentration in different samples (*p* < 0.05); a and b: significant difference with the same sample at different concentrations (*p* < 0.05). (**b**) Effect of PNPs on cell growth rate. The cell growth rate was determined from generations 1 to 10. Experiments were conducted in triplicate (n = 3), and the data are expressed as the mean ± SD. The data for the linear regression equation plotted by each group are as follows: y=−1.4x+417.5 (controls), y=−3.2x+362.8 (MG-induced), y=−1.9x+377 (PNP-40), y=−1.6x+382.1 (PNP-60), and y=−2x+384.7 (PNP-80). # Followed by a significant difference with the controls in the 10th generation (*p* < 0.05); * followed by significant difference with the MG-treated group in the 10th generation (*p* < 0.05).

Figure 8a shows the results, after 24 h of induction of Hs68 cells with MG at a concentration of 400 μM (Figure S1), the cell survival rate decreased to 75.6% compared to the controls. We found that the PNPs can protect the viability of MG-induced Hs68 cells. For PNP-40, PNP-60, and PNP-80, the cell viability was 84.4%, 86.2%, and 86.3% at a concentration of 0.5 mg/mL; 99%, 95%, and 100% at a concentration of 1.0 mg/mL; and 104.1%, 103.6%, and 104.6% at a concentration of 1.5 mg/mL, respectively. At 1 and 1.5 mg/mL, the nonsignificant difference with the controls showed that the PNPs exerted a protective effect on Hs68. At 1.5 mg/mL, the protective effect of PNPs was higher than at other concentrations. Accordingly, 1.5 mg/mL was selected for the subsequent experiments.

Figure 8b shows the linear regression equation plot for each group. The cell growth rate of the MG-induced group exhibited a larger decline compared to the controls. After pretreatment with PNPs, the cell growth rate slowed significantly, and the cell growth rate decreased significantly in the 10th generation. In the MG-induced group, the growth rate was 322 cells/h, while that for the controls was 397 cells/hour. The difference between these two groups was significant. PNP-40, PNP-60, and PNP-80 yielded respective Hs68 cell growth rates of 358, 364, and 363 cells/hour at the 10th generation. The significant difference compared to the MG-induced group indicated that the PNPs slowed the decline of cell growth activity caused by MG.

### 3.7. Effect of PNPs on MG-Induced ROS Production in Hs68 Cells

**Figure 9:**
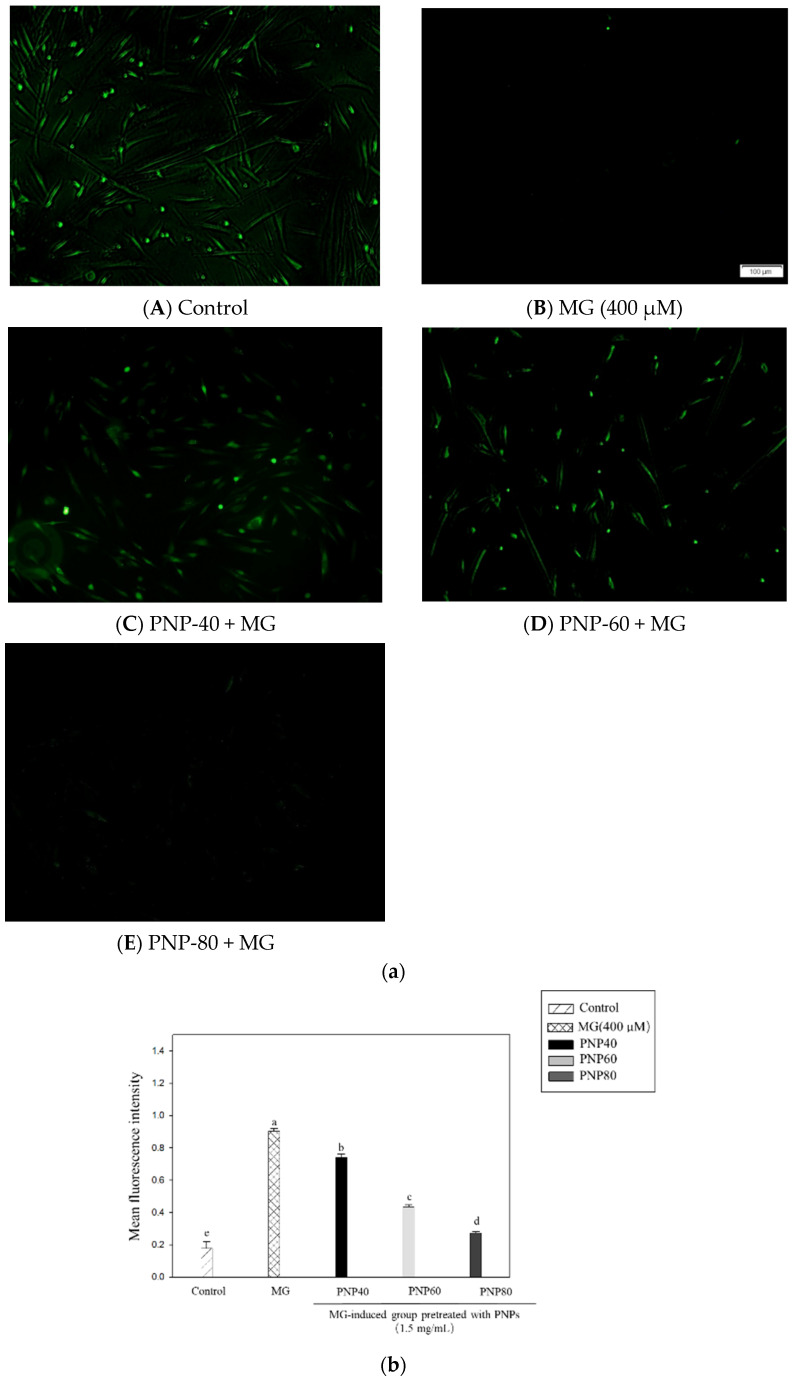
(**a**) Effect of MG-induced ROS generation in Hs68 cells. Scale bar: 100 μm. (**b**) Quantitative analysis of MG-induced Hs68 cell ROS production with or without PNPs pretreatment. ROS scavenging effect of PNPs (1.5 mg/mL) on Hs68 cells against MG-induced ROS generation obtained from the Image J analysis. a–e: Significant difference with different groups (*p* < 0.05).

In this experiment, the DCF-DA probe was used to determine the intracellular ROS in the MG-treated and PNP-treated groups. The results are shown in Figure 9. Compared with the controls, the fluorescence intensity of the group with MG (400 μM) increased to 5.1-fold, and the addition of PNPs significantly inhibited the generation of ROS. The inhibition rates of PNP-40, PNP-60, and PNP-80 were 18%, 52%, and 70%, respectively.

## 4. Discussion

In the early stage of glycation reaction, the amine group reacts with the complex reaction cascade caused by partial condensation of the reducing sugar α-hydroxy carbonyl group, leading to the formation of a Schiff base. Through a series of rearrangement reactions, the Schiff base produces Amadori products, a more stable Schiff base isomer [35]. The early glycation products have a relatively stable structure through oxidative degradation (i.e., glycoxidation), which is a critical reaction in the subsequent formation of AGEs [36]. The polysaccharide of *Misgurnus anguillicaudatus* have been found to exhibit antiglycation activity, with the highest inhibition rate of 37.3% being reported at a concentration of 200 μg/mL, which is higher than that of AG (16.3%) at the same concentration [31]. The polysaccharide BCP-1 extracted from the fruit of *R. nigrum* L. can also inhibit the formation of Amadori products during glycation. A previous study showed that different concentrations (0.025, 0.05, 0.10, 0.15, and 0.20 mg/mL) of BCP-1 exhibited inhibitory activity on the formation of Amadori products in a dose-dependent manner, with BCP-1 achieving the highest inhibitory activity (37.15%) at a concentration of 0.2 mg/mL [25]. The inhibitory effects of natural extracts on Amadori product formation were partly related to their chelation effects on metal ions [37]. Another study showed that the Fe^2+^-chelating ability of PNPs increased in a concentration-dependent manner, with the scavenging ability of PNP-80 reaching 97.75% at 5 mg/mL [13].

Carbonyl groups are produced when protein side chains are oxidized and are also produced in large quantities during glycation [38]. Protein carbonyl functional group content is a crucial indicator for evaluating protein oxidation and glycation. In a previous study on the antiglycation of kiwifruit (*Actinidia arguta*)-extracted polysaccharides, BSA and glucose were employed as glycation models, and the formation of protein carbonyl groups was significantly inhibited after introducing the exotic polysaccharides SPS-1, SPS-2, and SPS-3. At a concentration of 1 mg/mL, the respective inhibition rates were 42.6%, 55.6%, and 59.3%. Research has shown that in addition to the glycation process is the production of reactive oxygen and reactive nitrogen substances. These substances can oxidize the side chains of amino acid residues on proteins and form carbonyl derivatives, and the reduction of amine groups also aggravates protein glycation and damage under oxidative pressure. Therefore, the amine group in the protein is the main target in the glycation process, and the carbonyl content of the protein is the most common and commonly used biomarker for severe oxidative protein damage [39,40].

Reactive carbonyl species in the glycation process, such as 3-deoxyglucosone, glyoxal, and MG, play a critical role in mediating glycation stress in human cells and are associated with various chronic diseases and cancer [41,42]. Some studies have described MG as the most important precursor for the formation of AGEs during glycation. In animal experiments, MG-induced AGEs have been shown to cause cardiac dysfunction and myocardial infarction in mice. This may be due to MG modifying and interacting with collagen. The interaction of cells in the heart and blood vessels leads to cell apoptosis and decreased angiogenesis [43]. Studies have also claimed that MG, due to its highly reactive characteristics, can modify proteins in the human body or induce protein changes in secondary structures and the formation of toxic protein aggregates, such as amyloid, which can cause an imbalance of intracellular ion homeostasis, damage to the cell membrane, and lead to apoptosis [44].

Hs68 human fibroblasts are critical cells in the dermis of human skin. They are mainly responsible for synthesis and secretion. They play a key role in the formation of collagen [45,46]. Considerable evidence also indicates that MG is cytotoxic and can cause cell apoptosis, which plays a key role in the development of related diseases. MG can cause the formation of intracellular ROS and loss of mitochondrial membrane potential. It produces an endoplasmic reticulum stress response, which in turn leads to cell apoptosis [47]. The β-glucan in the polysaccharide may be the cause of cell proliferation. Previous studies have shown that β-glucan in the polysaccharide has the function of cell proliferation and promotes wound healing. Barley polysaccharides and black yeast (*Aureobasidium*) extract polysaccharides have been confirmed by animal and cell experiments as having the effect of making fibroblasts proliferate and promoting wound healing [48,49]. In PNP-40, PNP-60, and PNP-80, the β-glucan content was reported to be 20.20%, 12.20%, and 10.15% respectively [18]. Therefore, the cause of the increase in Hs68 cell activity may be the β-glucan contained in the polysaccharides.

Many studies have also explored the relationship between glycation and the accumulation of ROS. Some studies have proposed that the glycated products produced by dicarbonyl compounds, such as MG, malondialdehyde, and 4-hydroxy-2-nominal and macromolecules, play a key role in the cell’s response to oxidative stress [50]. In addition, the glycation response can activate the MAPK pathway, which is also related to the accumulation of ROS caused by glycation, in which the generation of ROS is a critical intermediate in the signal transduction of apoptosis. The overproduction of ROS caused by AGEs can induce apoptosis of fibroblasts, and the AGE-specific receptor (RAGE) may increase by activating NADPH oxidase due to the generation of ROS [51,52]. Therefore, we speculate that polysaccharides protect Hs68 cells to maintain cell viability under glycation stress due to their favorable antioxidant capacity and ability to scavenge intracellular ROS.

## 5. Conclusions

In the present study, we established an MG–BSA glycation model in vitro. The results showed that PNPs can significantly inhibit the production of AGEs in vitro. The antiglycation effect of PNP-80 is more favorable than that of PNP-40 and PNP-60. PNPs can increase the reduced cell viability and growth rate caused by glycation stress. Our results indicate that PNPs may protect cells from damage due to glycation stress through their excellent antioxidant effect and ROS scavenging ability. The antiglycation effect of PNPs is worthy of further research to discover its potential for mitigating damage caused by MG-induced glycation stress.

## Data Availability

Data are contained within the article or Supplementary Material.

## Supplementary Materials

The following are available online at https://www.mdpi.com/article/10.3390/antiox10101589/s1, Figure S1. Effect of MG on cell viability. Cells were pretreated with MG for (100–600 µM) for 24 h, and then their viability was determined using an MTT assay. Experiments were conducted in triplicate (n = 3), and the data are ex-pressed as the mean ± SD.
