# Supplementary material for: Effect of Pholiota nameko Polysaccharides Inhibiting Methylglyoxal-Induced Glycation Damage In Vitro
Source: Antioxidants (Basel). 2021 Oct 10;10(10):1589. doi: 10.3390/antiox10101589 (PMC8533542; doi:10.3390/antiox10101589)
Supplement: Supplementary file 1 [file antioxidants-10-01589-s001.zip › antioxidants-1403752-Supplementary Materials.pdf]

antioxidants

MDPI

Supplementary Materials

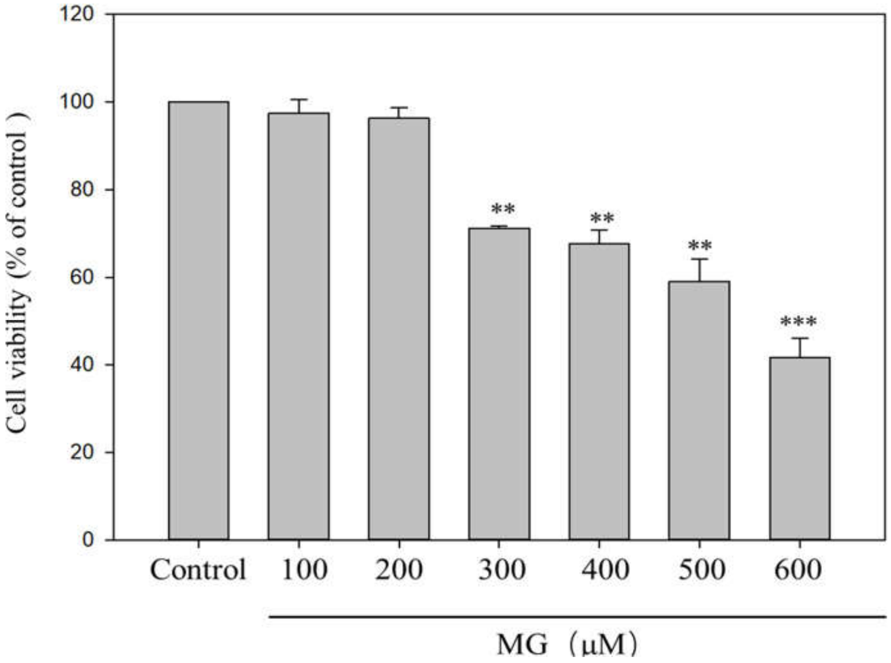

Figure S1. Effect of MG on cell viability. Cells were pretreated with MG for (100–600 μM) for 24 hours, and then their viability was determined using an MTT assay. Experiments were conducted in triplicate (n = 3), and the data are expressed as the mean ± SD. * Significant difference with the controls ($p < 0.05$); ** significant difference with the controls ($p < 0.01$); *** significant difference with the controls ($p < 0.001$).
